# Enhancing blue luminescence from Ce-doped ZnO nanophosphor by Li doping

**DOI:** 10.1186/1556-276X-9-480

**Published:** 2014-09-10

**Authors:** Qiang Shi, Changzheng Wang, Shuhong Li, Qingru Wang, Bingyuan Zhang, Wenjun Wang, Junying Zhang, Hailing Zhu

**Affiliations:** 1 School of Physical Science and Information Engineering, Liaocheng University, Shandong 252059, People's Republic of China; 2 Shandong Provincial Key Laboratory of Optical Communication Science and Technology, Shandong 252059, People's Republic of China; 3 School of Materials Science and Engineering, Liaocheng University, Shandong 252059, People's Republic of China; 4 Department of Physics, Beihang University, Beijing 100191, People's Republic of China; 5 Department of Physics and Optoelectronic Engineering, Weifang University, Weifang 261061, People's Republic of China

**Keywords:** (Li, Ce)-codoped ZnO, Blue luminescence, Phosphors, Sol-gel

## Abstract

Undoped ZnO, Ce-doped ZnO, and (Li, Ce)-codoped ZnO nanophosphors were prepared by a sol-gel process. The effects of the additional doping with Li ions on the crystal structure, particle morphology, and luminescence properties of Ce-doped ZnO were investigated by X-ray diffraction, scanning electron microscopy, X-ray photoelectron spectroscopy, electron paramagnetic resonance spectroscopy and photoluminescence spectroscopy. The results indicate that the obtained samples are single phase, and a nanorod shaped morphology is observed for (Li, Ce)-codoping. Under excitation with 325 nm light, Ce-doped ZnO phosphors show an ultraviolet emission, a green emission, and a blue emission caused by Zn interstitials. The spectrum of the sample codoped with a proper Li concentration features two additional emissions that can be attributed to the Ce^3+^ ions. With the increase of the Li doping concentration, the Ce^3+^ blue luminescence of (Li, Ce)-codoped ZnO is obviously enhanced, which results not only from the increase of the Ce^3+^ ion concentration itself but also from the energy transfer from the ZnO host material to the Ce^3+^ ions. This enhancement reaches a maximum at a Li content of 0.02, and then decreases sharply due to the concentration quench. These nanophosphors may promise for application to the visible-light-emitting devices.

**PACS:**

78.55.Et; 81.07.Wx; 81.20.Fw

## Background

ZnO is an *n*-type semiconductor material with a wide band gap of 3.3 eV and a large exciton binding energy of 60 meV. Room temperature photoluminescence (PL) spectra from ZnO can exhibit an ultraviolet (UV) emission and possibly one or more visible emissions caused by defects and/or impurities [1]. It has been reported that doping a ZnO host crystal structure with rare earth elements such as Tb, Er, and Ce can lead to excellent luminescence properties [2-4]. Especially, the blue light-emitting Ce-doped ZnO has received particular attention because of its high chemical stability, excellent optoelectronic properties, avirulence behavior, and biological compatibility, resulting in potential applications in the field of visible-light-emitting devices and biological fluorescence labeling [5]. Therefore, a further enhancement of the emission intensity in the blue emission band of Ce-doped ZnO phosphor is highly desirable.

It is well known that Li^+^ ions, as dopants, even in very small quantities, frequently play an important role in improving the luminescence intensity of phosphors. Gu et al. [6] reported that Li^+^ doping can enhance the luminescence of Dy-doped ZnO nanocrystals by increasing the recombination probability of electrons and trapped holes. Recently, Chen et al. [7] found that the red-light emission of a Eu-doped CaWO_4_ phosphor can be increased by using Li^+^ ions as charge compensators. A similar enhancement of the fluorescence of Pr-doped BaMoO_4_ phosphors via codoping with Li^+^ ions was obtained by He et al. [8]. Therefore, the incorporation of Li^+^ ions into a Ce-doped ZnO phosphor is also expected to enhance the blue luminescence intensity. To investigate this potential enhancement, we prepared samples of undoped ZnO, Ce-doped ZnO, and (Li, Ce)-codoped ZnO nanophosphors by a sol-gel process. Here, we focus on the effect of the variation of the concentration of Li^+^ ions on the structure, morphology, and luminescence properties of the Ce-doped ZnO phosphor, while the Ce doping concentration was kept at a constant level. Also, we discuss the origin of visible light emission in our samples and propose possible mechanisms to explain the enhanced blue luminescence caused by the codoping with Li^+^ ions. Our results demonstrate that (Li, Ce)-doped nanophosphors are promising candidates for applications in the field of visible-light-emitting devices.

## Methods

Undoped ZnO, Ce-doped ZnO, and (Li, Ce)-codoped ZnO phosphors were synthesized by a sol-gel process. Typically, the corresponding starting materials, Zn(CH_3_COO)_2_ · 2H_2_O, Ce(NO_3_)_3_ · 6H_2_O, and CH_3_COOLi · 2H_2_O, were mixed according to the nominal stoichiometric ratio (mol ratio, Zn/Ce/Li = (0.996 - *x*)/0.004/*x*, 0 ≤ *x* ≤ 0.04), and were dissolved in a certain amount of deionized water. Each solution was then added to 60 ml of a 1% (*w*/*v*) aqueous solution of polyvinyl alcohol acting as a stabilizer, and was stirred for 1 h. The mixtures were aged for 12 h at room temperature and then were heated to 80°C and maintained at this temperature until homogeneous gels had formed. The gels were air-dried at 120°C for 12 h, ground, and preheated at 400°C for 4 h in a muffle furnace. The last step consisted of a final annealing procedure at 550°C for another 4 h in air.

The samples thus obtained were investigated by X-ray diffraction (XRD) with CuK_α_ radiation (*λ* = 0.15406 nm) in order to identify the individual phases. The particle morphology was analyzed in a Hitachi S4800 (Hitachi, Tokyo, Japan) scanning electron microscope (SEM). PL spectra were recorded on an Edinburgh FLS920 spectrofluorometer (Edinburgh Instruments, Edinburgh, UK) equipped with a 450-W Xe lamp as the excitation light source. The X band electron paramagnetic resonance (EPR) spectra were determined by a Bruker ER-200D-SRC EPR spectrometer (Bruker, Billerica, MA, USA). X-ray photoelectron spectroscopy (XPS) experiments were performed on a Thermo ESCALAB 250XI multifunctional imaging electron spectrometer (Thermo Fisher Scientific, Waltham, MA, USA).

## Results and discussion

**Figure 1:**
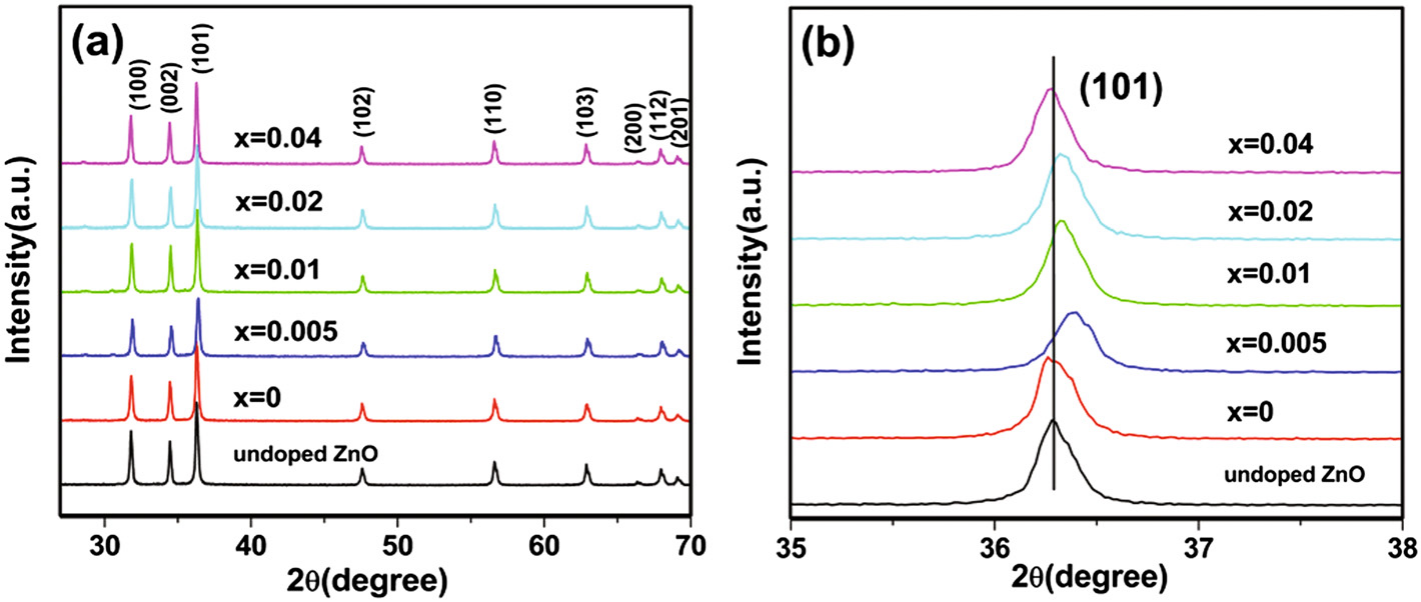
**XRD patterns of samples. (a)** XRD patterns of undoped ZnO and Ce_0.004_Zn_0.996 - *x*_O:*x*Li^+^ (*x* = 0, 0.005, 0.01, 0.02, 0.04) phosphors and **(b)** comparison of the corresponding (101) peaks.

Figure 1a shows the XRD patterns of undoped ZnO and Ce_0.004_Zn_0.996 - *x*_O:*x*Li^+^ (*x* = 0, 0.005, 0.01, 0.02, 0.04) phosphors annealed in air at 550°C for 4 h. The observed XRD reflections match the standard diffraction pattern of ZnO (JCPDS no. 80-0075). No diffraction peaks from other phases have been detected, indicating that the samples obtained are single phase and the Ce and codoped Li ions incorporated into the ZnO lattice do not change the crystal structure of ZnO. As can be seen from Figure 1b, for Ce_0.004_Zn_0.996_O, the position of the (101)-peak is shifted slightly to a lower angle compared to undoped ZnO, indicating that the lattice parameters of Ce_0.004_Zn_0.996_O are a slightly larger than those of undoped ZnO. The variation of lattice parameters demonstrates that Ce ions are incorporated into the ZnO lattice. It is expected that the substitutional doping of Ce ions for Zn ions would increase the lattice parameter of ZnO because the ionic radius of Ce^3+^ (0.103 nm) and Ce^4+^ (0.092 nm) is bigger than that of Zn^2+^ (0.074 nm). In addition, interstitial Ce could also cause the lattice parameter to expand [9]. According to George et al. [9], at low Ce concentrations, interstitial incorporation is favored, while at high Ce concentrations, substitution and interstitial substitution are comparable processes. Therefore, we suggest that interstitial Ce plays a predominant role in the Ce-doped ZnO crystal for a Ce concentration of 0.004. In the (Li, Ce)-codoped ZnO sample, when Li^+^ ions substitute into the Zn^2+^ ions, the lattice parameters will be decreased due to the smaller ionic radius of Li^+^ (0.068 nm) compared to that of Zn^2+^. Therefore, the (101)-peak shifts towards a larger angle from 36.26° to 36.40°, as the Li doping concentration (*x*) is changed from 0 to 0.005. However, as the Li concentration is then further increased, the position of the (101)-peak shifts back towards a smaller angle. This indicates that there is another competing process at work which increases the lattice parameters. A similar variation has been observed in Li-doped ZnO films, in which the abnormal shift in the 2*θ* value with an increase in Li content was attributed to the presence of interstitial Li ions in the Li-doped ZnO film [10]. It is well known that the incorporation of Li ions into the ZnO lattice may cause the formation of substitutional and/or interstitial Li ions. In our experiments, we suggest that substitutional Li ions play a predominant role in the (Li, Ce)-codoped ZnO crystal for *x* ≤ 0.005, whereas for *x* ≥ 0.01, interstitial Li ions play a predominant role. Thus, we believe that the decrease of the lattice parameters is attributed to substitutional Li ions, while the increase is due to interstitial Li ions. Therefore, the XRD results show that Li doping actually has a great influence on the structure of Ce-doped ZnO phosphors.

**Figure 2:**
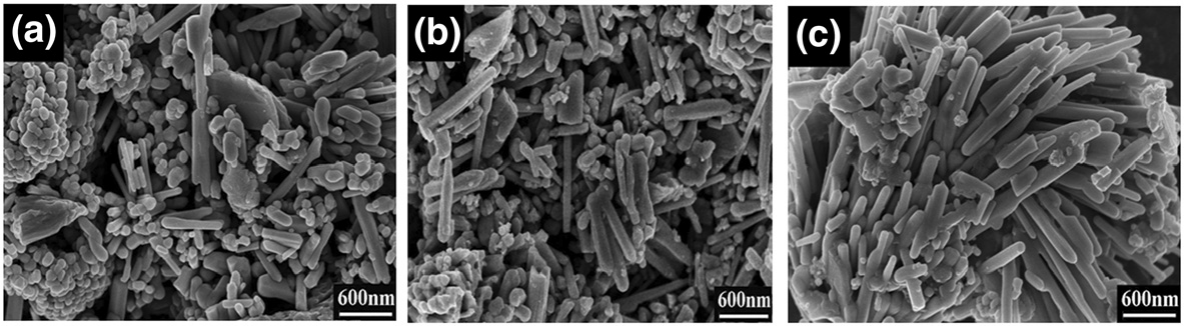
**SEM images of samples.** SEM images of **(a)** undoped ZnO, **(b)** Ce_0.004_Zn_0.996_O and **(c)** Ce_0.004_Zn_0.996-x_O:xLi^+^ (x = 0.02) phosphors.

The morphology of undoped ZnO, Ce_0.004_Zn_0.996_O and Ce_0.004_Zn_0.996 - *x*_O:*x*Li^+^ (*x* = 0.02) phosphors annealed in air at 550°C for 4 h was studied by SEM. Figure 2a shows that the undoped ZnO particles are composed of granules and rods. A similar morphology can be seen in Figure 2b, indicating that the incorporation of Ce ions into the ZnO lattice does not change the overall morphology. However, the morphology of Ce_0.004_Zn_0.996 - *x*_O:*x*Li^+^ (*x* = 0.02) phosphors is apparently different. As Figure 2c reveals, the particles are now mostly rod-shaped and the granular particles have almost disappeared. The average diameter of these rods is about 100 nm. The results of the SEM investigation illustrate that the growth of rods may be promoted by Li doping.

**Figure 3:**
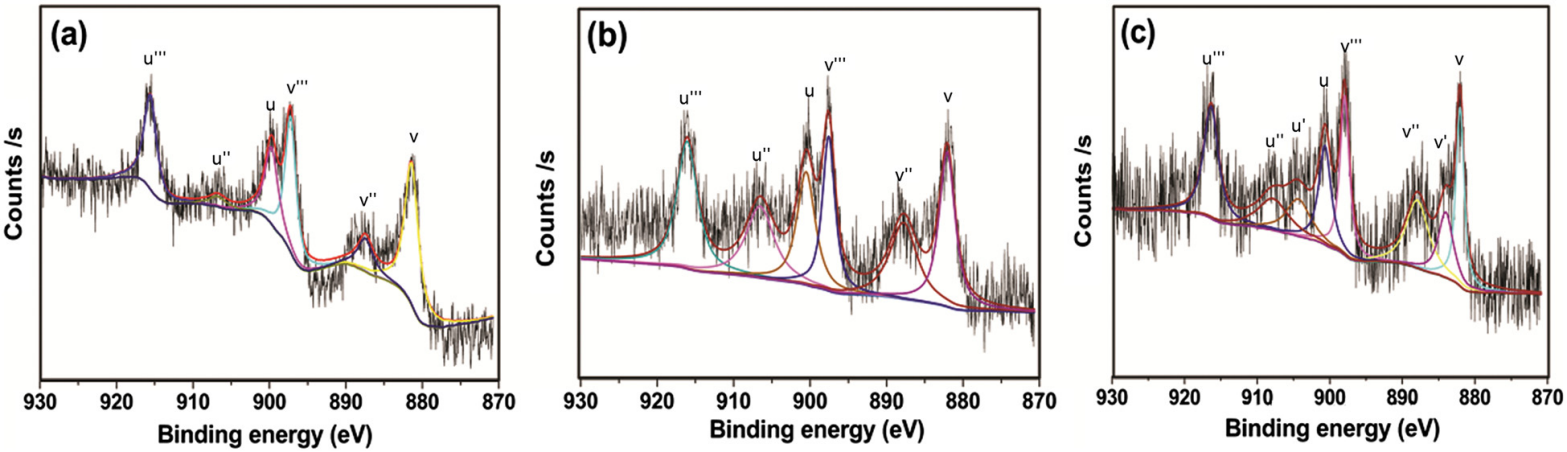
**Core level XPS of Ce3d.** Core level XPS of Ce3d for **(a)** Ce_0.004_Zn_0.996_O, **(b)** Ce_0.004_Zn_0.996 - *x*_O:*x*Li^+^ (*x* = 0.005) and **(c)** Ce_0.004_Zn_0.996 - *x*_O:*x*Li^+^ (*x* = 0.02) phosphors.

It is well known that the element Ce has two different oxidation valence states, Ce^4+^ and Ce^3+^. To identify the oxidation state of Ce in our samples, XPS spectra of Ce_0.004_Zn_0.996 - *x*_O:*x*Li^+^ (*x* = 0, 0.005, 0.02) phosphors were measured and are shown in Figure 3. As shown in Figure 3a,b the Ce3d XPS spectra of Ce_0.004_Zn_0.996_O and Ce_0.004_Zn_0.996 - *x*_O:*x*Li^+^ (*x* = 0.005) phosphors both exhibit six binding energy peaks labeled as *v*, *v″*, and *v*‴ and *u*, *u*″, and *u*‴, where *u* and *v* represent the two different spin orbit (3d_5/2_ and 3d_3/2_) contributions. The three doublets of the spin-orbit split components are assigned to Ce^4+^ final states because similar results have been observed in the Ce3d XPS spectra of Ce^4+^ compounds [11-13]. The peaks labeled as *v* and *u* are attributed to the final state of Ce 3d^9^ 4f^2^ O 2p^4^, while *v*″, *u*″ and *v*‴, *u*‴ can be assigned to the Ce 3d^9^ 4f^1^ O 2p^5^ and Ce 3d^9^ 4f^0^ O 2p^6^ final states, respectively [13]. No signals connected to Ce^3+^ were observed in Figure 3a,b. This probably indicates that the Ce^3+^ compound is either amorphous or has a very low concentration in Ce_0.004_Zn_0.996 - *x*_O:*x*Li^+^ (*x* = 0, 0.005) phosphors [13]. In Figure 3c, besides the six peaks assigned to Ce^4+^, additional peaks located at 884.1 and 904.3 eV (labeled as *v*′ and *u*′, respectively) can be observed, which can be assigned to the Ce 3d^9^ 4f^1^ O 2p^6^ of the Ce^3+^ state by comparison with data from literatures [12-14], meaning that Ce^3+^ and Ce^4+^ coexist in Ce_0.004_Zn_0.996 - *x*_O:*x*Li^+^ (*x* = 0.02) phosphors. XPS quantitative analysis of Ce_0.004_Zn_0.996 - *x*_O:*x*Li^+^ (*x* = 0.02) phosphors is given in Table 1. The peak area percentage can be used to determine the relative concentration, and it can be calculated from the ratio between the area of the peak and the total area in the Ce 3d region. The obtained values are listed in Table 1. From this table, we can see that the relative total concentration of the Ce^4+^ is 81.8% while that of the Ce^3+^ is 18.2% and the atomic ratio between Ce^3+^ and Ce^4+^ is equal to 0.22. These results indicate that the incorporation of Li ions into the ZnO lattice may lead to the reduction of Ce^4+^ to Ce^3+^. From XRD results, we can see that interstitial Li ions play a predominant role for *x* ≥ 0.01. In general, interstitial Li ions acting as electron donors will result in a higher electron density. Thus, Ce^4+^ ions have more opportunities to trap electrons and are converted into Ce^3+^ ions. Therefore, we believe that incorporating Li ions with sufficient concentration in Ce-doped ZnO can reduce Ce^4+^ to Ce^3+^ without the need for a heat treatment and the reduction of Ce^4+^ to Ce^3+^ is due to the interstitial Li ions, which is in good agreement with the results reported by Renaudin et al. [15].

**Table 1:** **XPS quantitative analysis of Ce**_**0.004**_**Zn**_**0.996-x**_**O:xLi**^**+ **^**(x = 0.02) phosphors**

		**Spin-orbit**	**Position**	**Area (%)**	**Total area (%)**
Ce^4+^	v	3d_5/2_	882.1	14.4	81.8
	u	3d_3/2_	900.7	11.9	
	v″	3d_5/2_	887.9	14.9	
	u″	3d_3/2_	907.8	9.3	
	v‴	3d_5/2_	897.9	14.8	
	u‴	3d_3/2_	916.4	16.5	
Ce^3+^	v′	3d_5/2_	884.1	9.5	18.2
	u′	3d_3/2_	904.3	8.7	

**Figure 4:**
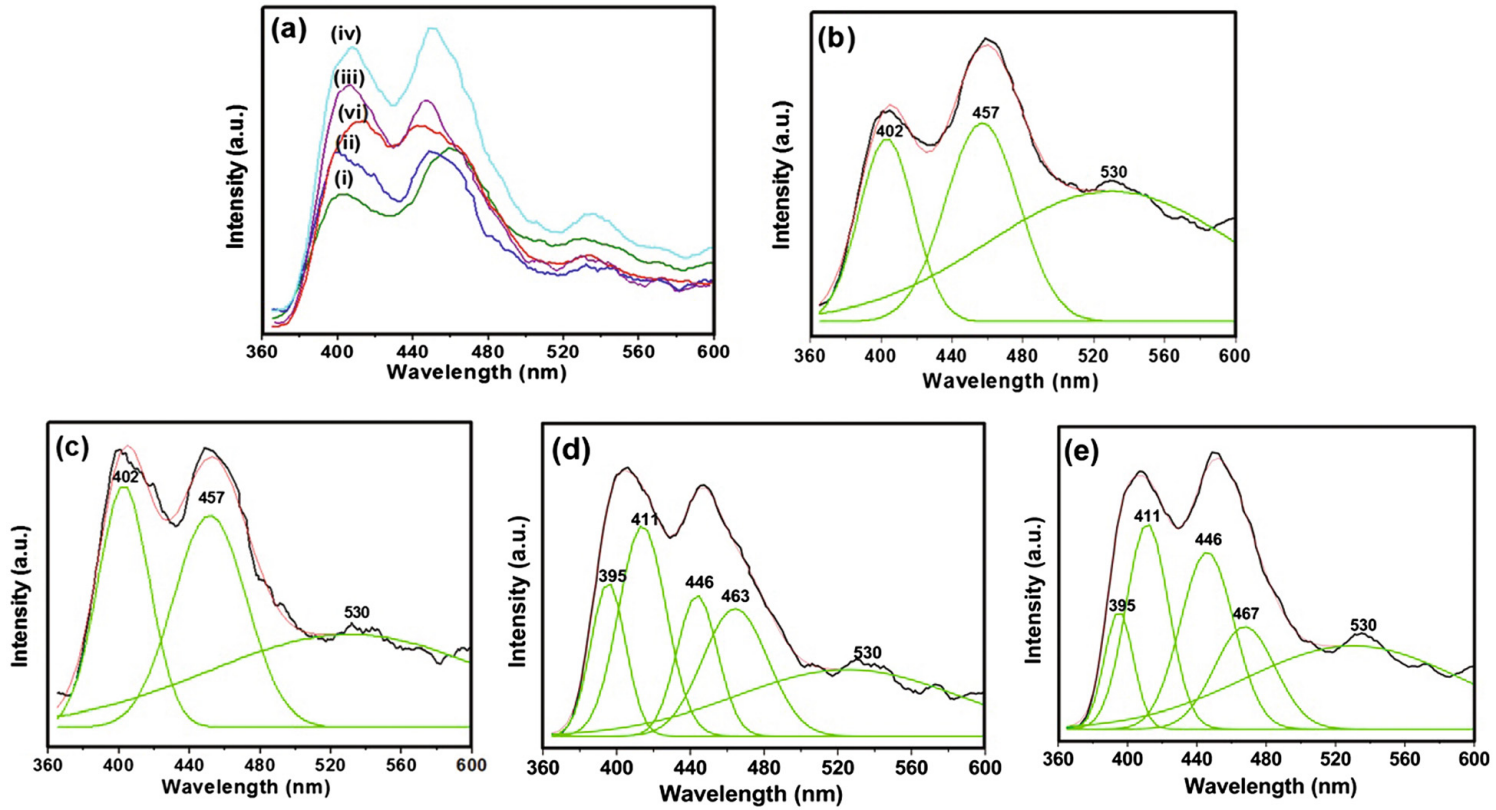
**PL spectra of samples. (a)** PL spectra of Ce_0.004_Zn_0.996 - *x*_O:*x*Li^+^ with *x* = 0 (i), *x* = 0.005 (ii), *x* = 0.01 (iii), *x* = 0.02 (iv), and *x* = 0.04 (v). **(b)**, **(c)**, **(d)**, and **(e)** are the corresponding Gaussian curve fits for the spectra i, ii, iii, and iv in **(a)**.

PL spectra of Ce_0.004_Zn_0.996 - *x*_O:*x*Li^+^ (*x* = 0, 0.005, 0.01, 0.02, 0.04) phosphors were measured with the excitation wavelength fixed at 325 nm (Figure 4). As shown in Figure 4a, the PL spectra of all samples consist of a UV emission assigned to the near band edge transition of ZnO and several visible emissions due to defects and/or dopants. It is worthwhile to note that with the increase of Li doping concentration, the blue emission band intensity increases, reaching a maximum at about *x* = 0.02 and then decreases at a higher doping concentration. The effect of Li doping on the blue-light emission is very interesting and will be further discussed below. Figure 4b,c,d,e represents the corresponding Gaussian fits for the spectra i, ii, iii, and iv in Figure 4a. It can be seen that the positions of the UV emission from Ce_0.004_Zn_0.996 - *x*_O:*x*Li^+^ (*x* = 0, 0.005, 0.01, 0.02) phosphors are located at 402, 402, 395, and again 395 nm, respectively. When Ce and Li ions are incorporated into the ZnO lattice, the bandgap structure of ZnO is modulated, causing the difference in the UV emission [16]. The most interesting observation is that, for the Ce_0.004_Zn_0.996_O and the Ce_0.004_Zn_0.996 - *x*_O:*x*Li^+^ (*x* = 0.005) samples, the positions of the blue-light emission peaks are both located at about 457 nm, whereas for the Ce_0.004_Zn_0.996 - *x*_O:*x*Li^+^ (*x* = 0.01, 0.02) samples, the blue emission bands can be modeled by Gaussian fits as three peaks centered at the positions of about 411, 446, and 463 nm (467 nm) respectively.

**Figure 5:**
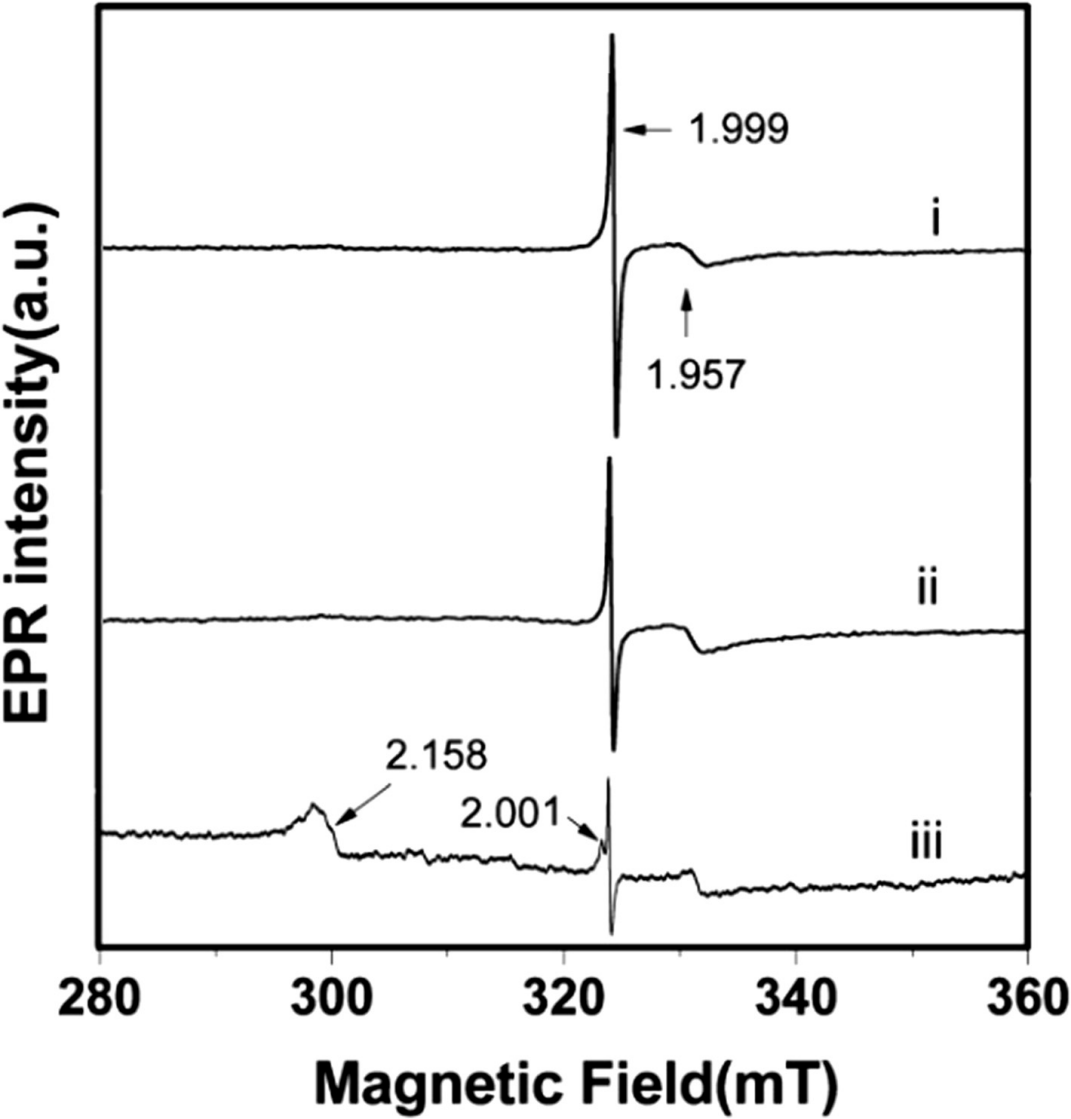
**EPR spectra of samples.** EPR spectra of Ce_0.004_Zn_0.996 - *x*_O:*x*Li^+^ with *x* = 0 (i), *x* = 0.005 (ii), and *x* = 0.02 (iii), respectively.

In order to study the origin of these visible emissions, we measured the EPR spectra of Ce_0.004_Zn_0.996 - *x*_O:*x*Li^+^ (*x* = 0, 0.005, 0.02) phosphors (Figure 5). Figure 5i and ii both show two EPR signals at *g* = 1.999 and *g* = 1.957, respectively. The low-field signal at *g* = 1.999 is generally attributed to unpaired electrons trapped in oxygen vacancies [5,17], while the high-field signal at *g* = 1.957 is ascribed to the Zn interstitial site. In the present work, as seen in Figure 4b,c, for the Ce_0.004_Zn_0.996 - *x*_O:*x*Li^+^ (*x* = 0.005) phosphors, the ratio of emission intensities of green (530 nm) to blue (457-467 nm) is larger than that of the Ce_0.004_Zn_0.996_O phosphors. Also, the same variation of intensity ratio of signals at *g* = 1.999 to 1.957 is shown in Figure 5i and ii, indicating that the two signals at *g* = 1.999 and *g* = 1.957 can be related to the green emission (530 nm) and the blue emission (457 to 467 nm), respectively. Therefore, we suggest that oxygen vacancies are responsible for the green emission (530 nm), while the blue emission (457 to 467 nm) is due to Zn interstitial. These results are in agreement with previously reported results [5,18-20].

Figure 5iii depicts the EPR spectrum of the Ce_0.004_Zn_0.996 - *x*_O:*x*Li^+^ (*x* = 0.02) sample. In addition to the two signals at *g* = 1.999 and *g* = 1.957, two additional signals at *g* = 2.158 and *g* = 2.001 are clearly visible. According to literatures [21,22], the weaker signal at *g* = 2.001 can be attributed to O_2_^-^ adsorbed on the sample surface. From the XPS results, we know that Ce^3+^ and Ce^4+^ coexist in Ce_0.004_Zn_0.996 - *x*_O:*x*Li^+^ (*x* = 0.02) phosphors. Ce^4+^ ions are diamagnetic and cannot be detected by the EPR technique. On the other hand, Ce^3+^ ions are paramagnetic, with spin 1/2, and thus can be detected. It has been reported that Ce^3+^ ions located in an axisymmetrical field are characterized by the EPR signal pair *g*_∥_ = 1.94 and *g*_⟂_ = 1.96, while Ce^3+^ ions in different asymmetric fields give different resonance signals [22-24]. In our work, Figures 1b and 5iii confirm the presence of interstitial Li, substitutional Li, interstitial Zn, and oxygen vacancies in the Ce_0.004_Zn_0.996 - *x*_O:*x*Li^+^ (*x* =0.02) crystal, which could destroy the axial symmetry of the field around Ce^3+^ and lead to Ce^3+^ ions located in asymmetric sites. Also, Wang et al. [24] reported a broad signal of *g* = 2.15 for Ce(OH)_3_, which is a typical EPR signal of Ce^3+^ in Ce(OH)_3_ nanorods. Therefore, we ascribe the signal at *g* = 2.158 to Ce^3+^ being in an asymmetric field. In addition, the energy difference between the two emission peaks (411 and 446 nm) is about 1,910 cm^-1^, which is very close to the theoretical difference of about 2,000 cm^-1^ between the ^2^F_5/2_ and ^2^F_7/2_ ground state levels of Ce^3+^[25]. Thus, based on the analysis above, we believe that the blue emissions at about 411 and 446 nm are associated with the respective ^2^D_3/2_ → ^2^F_5/2_ and ^2^D_3/2_ → ^2^F_7/2_ transitions of Ce^3+^.

**Figure 6:**
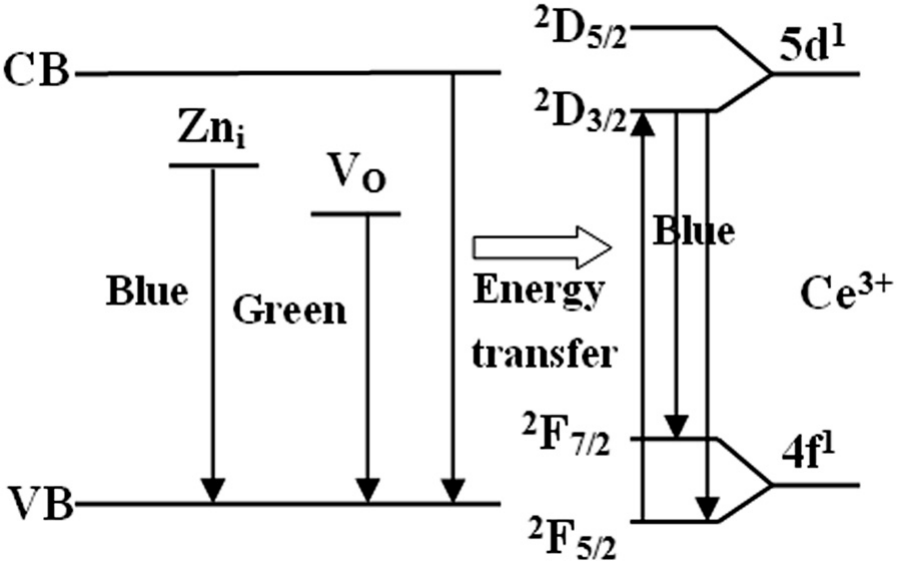
Energy-level diagram of (Li, Ce)-codoped ZnO.

The intensities of the Ce^3+^ blue emissions are higher than the emissions for intrinsic ZnO (Figure 4d,e), indicating that an energy transfer is more likely to take place from the ZnO host to Ce^3+^. The ZnO host absorbs energy from the excitation source resulting in the creation of excitons. Subsequently, a part of the recombination energy can be transferred to the Ce^3+^ ions through a resonant energy transfer process, which promotes the excitation from the ground 4f states to the excited 5d states on the Ce^3+^ center [26]. Therefore, subsequent radiative relaxation would result in enhanced blue-light emissions of Ce^3+^. The energy transfer from ZnO to Ce^3+^ has also been observed in Ce-doped ZnO thin films [26] and Ce-doped ZnO-SiO_2_ powders [27]. To better understand the possible transitions in Ce^3+^ and the energy transfer from the ZnO host, an energy-level diagram of (Li, Ce)-codoped ZnO is shown in Figure 6. In addition, from the XRD and XPS results, we can conclude that with the increase of Li doping concentration, more Li ions occupy the interstitial sites, and thus Ce^4+^ ions have more opportunities to be reduced to Ce^3+^ ions, which results in the increase of the Ce^3+^ ion concentration. Therefore, the Ce^3+^ blue luminescence of (Li, Ce)-codoped ZnO is obviously gradually enhanced as the Li concentration increases, which results not only from the energy transfer from the ZnO host to Ce^3+^ but also from the increase of the Ce^3+^ ion concentration, reaching a maximum at *x* = 0.02. It then decreases sharply due to the concentration quench as the Li concentration further increases. In addition, a red shift of the blue emission assigned to Zn interstitial can be observed as the increase of Li doping concentration. The shift is likely due to defect ionization, resulting in the formation of extended Zn interstitial states, which are below the simple Zn interstitial state [5].

## Conclusions

In summary, undoped, Ce-doped and (Li, Ce)-codoped ZnO phosphors were synthesized using a sol-gel process. The crystal structure, particle morphology, and luminescence properties of the obtained samples were investigated as a function of the content of Li ions. All of the samples only show a single phase. For a Li content of *x* ≤ 0.005, substitutional Li ions play a predominant role in the (Li, Ce)-codoped ZnO crystal, while interstitial Li ions play a predominant role for *x* ≥ 0.01. PL spectrum of Ce-doped ZnO consists of a UV emission, a blue emission related to the Zn interstitial, and a green emission assigned to oxygen vacancies. Comparing these results with the PL spectra for (Li, Ce)-codoped ZnO (*x* ≥ 0.01) phosphors, in addition to the blue emission due to the Zn interstitial (at 463 to 467 nm), the latter exhibit two additional strong blue emissions at 411 and 446 nm ascribed to the Ce^3+^ ions. This is because incorporating Li ions at a sufficient concentration in Ce-doped ZnO can cause the reduction of Ce^4+^ to Ce^3+^. As the Li doping concentration is increased, the intensity of the blue-light emissions related to Ce^3+^ increases, which results not only from the increase of the Ce^3+^ ion concentration itself but also from the energy transfer from the ZnO to Ce^3+^. It reaches maximum at about *x* = 0.02, and then decreases sharply at a higher doping concentration due to the concentration quench. (Li, Ce)-codoped ZnO phosphors are expected to find potential applications in the field of visible-light-emitting devices.

## Competing interests

The authors declare that they have no competing interests.

## Authors' contributions

QS and CW developed the concept and design of the (Li, Ce)-codoped ZnO nanophosphors. QS carried out the experiments and drafted the manuscript. SL, QW, and BZ participated in the preparation and characterization of the (Li, Ce)-codoped ZnO nanophosphors. WW, JZ, and HZ participated in the design and the discussion of this study. All authors read and approved the final manuscript.
